# HDACs/mTOR inhibitor synergizes with pyrotinib in HER2-positive pancreatic cancer through degradation of mutant P53

**DOI:** 10.1186/s12935-022-02807-4

**Published:** 2022-12-01

**Authors:** Tiebo Mao, Xiaofei Zhang, Haiyan Xu, Xiao Zhang, Weiyu Ge, Shumin Li, Jingyu Ma, Ming Yue, Shengbai Xue, Jiujie Cui, Liwei Wang

**Affiliations:** State Key Laboratory of Oncogenes and Related GenesDepartment of OncologySchool of Medicine, Shanghai Cancer InstituteRenji HospitalShanghai Jiao Tong University, Shanghai, China

**Keywords:** Pancreatic ductal adenocarcinoma, Pyrotinib, HER2, HDAC, mTOR, Apoptosis

## Abstract

**Background:**

Pancreatic ductal adenocarcinoma (PDAC), as a highly lethal malignancy with high mortality, lacks of effective treatment. Canonical therapeutic targets in PDAC demand further verification among which HER2 receptor tyrosine kinase inhibitor pyrotinib as treatment targets has not be decided.

**Methods:**

Anti-PDAC efficacy of pyrotinib was evaluated both in vitro and in vivo using both cell lines and patient-derived xenografts. By screening a large-scale library of 1453 compounds, we identified HDACs/mTOR inhibitor 1 as a promising candidate to synergize with pyrotinib. The combination therapy was evaluated in vitro and in vivo in multiple cell lines and animal models. Furthermore, RNA-seq analysis was performed to reveal the latent molecular mechanism of combination therapy.

**Results:**

In our study, pyrotinib monotherapy was found to be inefficient to anti-PDAC which exhibited limited anti-proliferation effect in vitro and in vivo. Through therapy combined with HDACs/mTOR inhibitor 1, pyrotinib triggered intense apoptosis in PDAC both in cell lines and animal models. Mechanistic analyses revealed that mutant P53 degradation mediated by HDAC inhibition synergized with HER2 and mTOR inhibition.

**Conclusions:**

In conclusion, identification of HDACs/mTOR inhibitor as a synergistic inhibitor, provides a potent therapeutic strategy that targets HER2-positive pancreatic cancer.

**Supplementary Information:**

The online version contains supplementary material available at 10.1186/s12935-022-02807-4.

## Introduction

Pancreatic ductal adenocarcinoma (PDAC), is a highly lethal cancer with a median survival of less than 10 months after diagnosis and a 5-year survival rate below 10% [1]. Concerning that over 50% of tumors are diagnosed with systemic metastases, systemic therapy like chemotherapy is the main strategy against PDAC but limited choices of regimens tremendously impair the prognosis of patients. Although PDAC is universally acknowledged barren concerning targeted therapy, exploration for potential actionable targets never ends in decades. Different types of monoclonal antibodies such as trastuzumab and pertuzumab and tyrosine kinase inhibitors (TKIs) that failed in overwhelming PDAC call us to look into combination treatment that may correspond with its complex multi-step genetic alterations [2].

The human epidermal growth factor receptor 2 (*ERBB2, HER2*) gene encodes for a transmembrane protein which operates as a growth factor receptor tyrosine kinase. It regulates cell proliferation, apoptosis, differentiation and angiogenesis. The activated HER2 stimulates various downstream signaling pathways including RAS/RAF/MEK/ERK pathway and PI3K/AKT pathway. Gene amplification of *HER2* and overexpression of HER2 protein have been well studied in breast cancer and provide treatment choices for around 15 to 20% of patients harboring these features [3]. As a counterpoint to breast cancer, strategy of the inhibition of overexpressed HER2 in pancreatic cancer has met failure or awaited further exploration in clinical trials [4–6]. Furthermore, reported studies on HER2 expression status in PDAC varies widely (21 to 61%) due to different staging and methodology [7–11]. In this study, we explored the expression patterns of HER2 in our PDAC cohort. Concerning that the efficacy of TKIs targeting HER2 in PDAC remains obscure, we then took explorations of pyrotinib against PDAC using both pancreatic cancer cell lines and patient-derived xenografts (PDX).

Pyrotinib which is different from trastuzumab, is an oral, irreversible pan-HER receptor TKI which mainly act against HER2 (Additional file 1: Table S1). Its efficacy of inhibiting proliferation and metastasis of HER2-overexpressing cells in vitro and in vivo has been proved in preclinical data together with phase I/II clinical trials in breast cancer, gastric cancer and non-small-cell lung cancer [12–15]. However, the efficacy of pyrotinib against HER2-overexpression PDAC is yet not decided. After elucidating the expression patterns of HER2 in PDAC, we demonstrated the efficacy of pyrotinib on HER2-overexpression PDAC both in vitro and in vivo. Furthermore, latent agents for synergistic combination were screened with expectation of extraordinary toxicity on PDAC.

Through the large scale screening here we report a HDACs/mTOR inhibitor screened in a large scale small-molecular library that shows high level of synergistic effect as well as toxicity combined with pyrotinib in PDAC [16]. We reveal that HDACs/mTOR inhibitor 1 promotes the degradation of gain-of-function mutant P53 via inhibiting HDAC6 and thus exacerbate apoptosis triggered by pyrotinib. As one of the most frequently mutated gene in PDAC, P53 is a preferred target [17, 18]. Mutation of P53 in PDAC frequently results in the loss of functions(LOFs) necessary for tumor suppression and even gain of functions(GOFs) that may promote tumor growth as well as apoptosis resistance [19, 20]. HDAC6 inhibition cancer abort the stability and accumulation of mutant P53 through disrupting HDAC6/Hsp90/mutp53 chaperone [21]. The combined inhibition of HER2 and mTOR provides a promising therapeutic strategy in HER2 positive PDAC.

## Materials and methods

### Human PDAC tissue array analysis

The study was conducted in accordance with International Ethical Guidelines for Biomedical Research Involving Human Subjects (CIOMS). The study was approved by the Research Ethics Committee of Ren Ji Hospital, School of Medicine, Shanghai Jiao Tong University (Shanghai, P.R. China). Written informed consent was provided to all the patients before enrollment. The patient cohort of human pancreatic tissue array containing 90 PDAC specimens and corresponding noncancerous tissues were also obtained from Ren Ji Hospital (Shanghai Jiao Tong University School of Medicine, Shanghai, P.R. China) from January 2010 to January 2018. Patients had not received radiotherapy, chemotherapy, or other related antitumor therapies before surgery. Before surgery, none of the patients had received antitumor therapies. The tissue staining was scored 0 when < 5% tumor cells showed expression. Positive scores (1–3) were based on percent of tumor cells and staining intensity within the tumor sample.

### Xenograft studies

Female BALB/c nude mice of 6–8 weeks old were manipulated and raised according to protocols approved by the Shanghai Medical Experimental Animal Care Commission. For the cell-derived xenograft model, 2*10^6^ AsPC-1 cells were suspended in 200 μl PBS and subcutaneously injected into the upper flank side of each mouse (n = 6). For the patient-derived xenograft model, biopsy samples were obtained from patients with early-stage pancreatic cancer who underwent surgeries and then planted subcutaneously into the flank of nude mice. Implantation of subsequent passages provided stable and comparable xenografts, and fragments (3*3*3 mm^3^) in 4th or 5th passage of PDXs were then surgically implanted. Once tumors, CDXs or PDXs, grew to approximately 100mm^3^ they were randomized for treatment. In the monotherapy treatment, pyrotinib was delivered orally every day with 40 mg/kg or 80 mg/kg for 21 days. Trastuzumab (an anti-HER2 monoclonal antibody) was given through intraperitoneal injection twice a week with 10 mg/kg for 21 days. In the combination therapy treatment, control group-mice received intraperitoneally injection of 100 μl PBS every 2 days and 200 μl vehicle orally every day; monotherapy group-mice received intraperitoneally injection of 100 μl PBS every 2 days and pyrotinib with 20 mg/kg orally every day or 10 mg/kg HDACs/mTOR inhibitor 1 every 2 days and 200 μl vehicle orally every day; combination-therapy group mice received intraperitoneally injection of 10 mg/kg HDACs/mTOR inhibitor 1 every 2 days and pyrotinib with 20 mg/kg orally every day. The mice’s health status was monitored every 2 or 3 days for weight changes or for signs of altered motor while in their cages. At the end of study, mice were euthanized according to approved SMEACC protocol. Tumors from all animals were excised and embedded in paraffin for immunohistochemical analyses.

### Statistical analysis

All descriptive statistics were presented as the mean ± standard deviation (SD) or standard error of mean (SEM). Analyses of variance models, student's t-tests, chi-square tests were used to compare mean values between the test and control samples. Statistical analyses were performed with IBM SPSS version 22 (SPSS, Armonk, NY, USA) and GraphPad Prism version 8. Results were presented as the mean ± SD or SEM of three independent tests. The statistical significance of the difference between test and control samples was assessed at significance thresholds of *P < 0.05, **P < 0.01, ***P < 0.001 and ****P < 0.0001.

## Results

### HER2 is overexpressed in PDAC

**Fig. 1:**
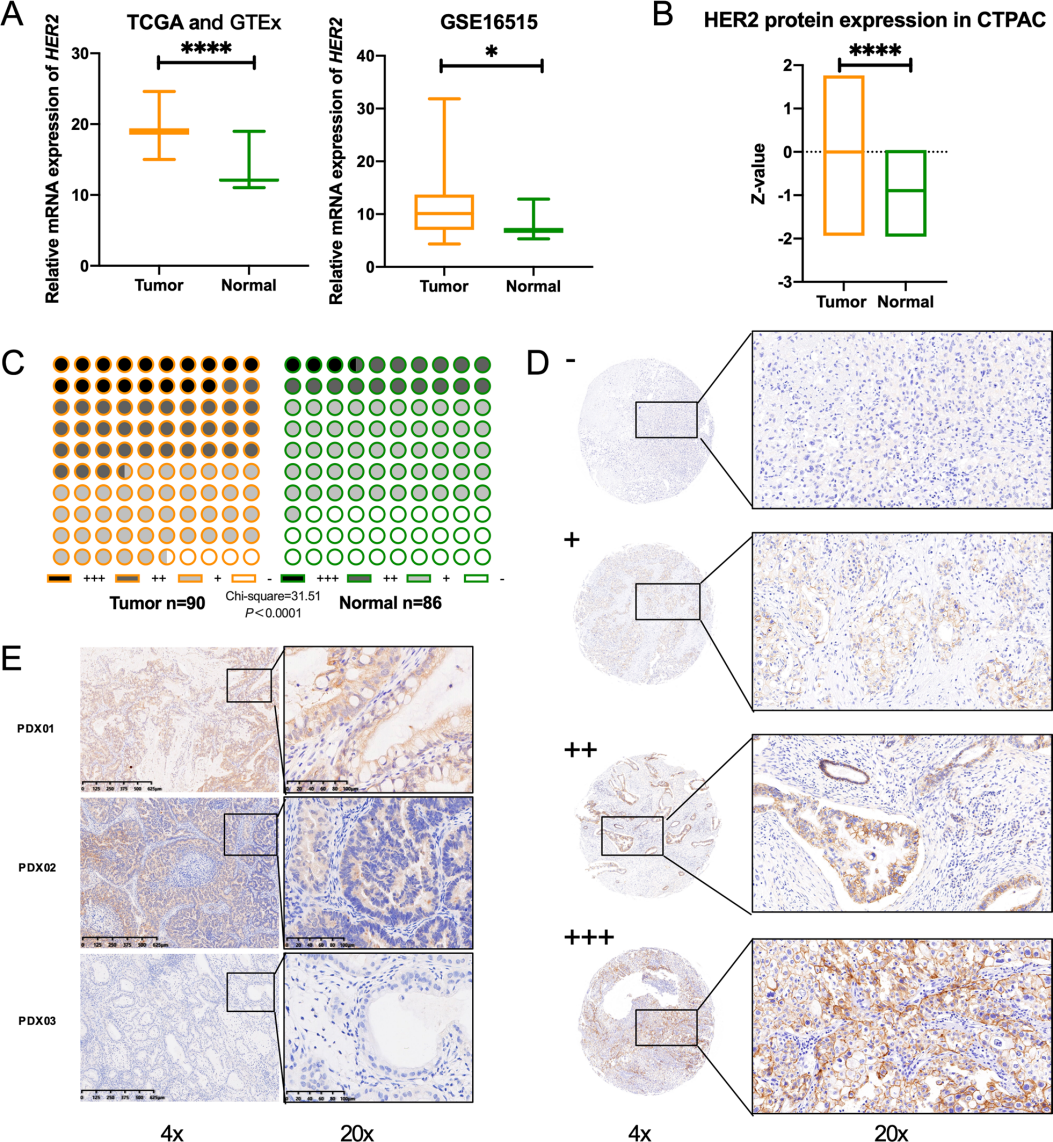
Up-regulation of HER2 in PDAC. **A**, **B** *HER2* gene expression and protein expression in samples of tumor and adjacent normal tissue from patients with PDAC in the TCGA (left) or GSE (right) cohorts and CTPAC database. **C** IHC score scaled from – to +  +  + evaluated independently in tissue microarray of Renji cohort between tumor and paired adjacent normal tissue. **D**, **E** Representative IHC staining for HER2 performed in tissue microarray of Renji cohort (**D**) and PDX models (passage 5th) (**E**). IHC score scaled from −  to +  +  + indicates negative, low, medium and high expression of HER2. Statistical tests: unpaired two-tailed Student t test in (**A**, **B**), chi-square test in (**C**). *P < 0.05, **P < 0.01, ***P < 0.001, ****P < 0.0001

In the attempt to reveal the expression pattern of HER2 in PDAC, we firstly analyzed the expression of HER2 in PDAC using TCGA, GTEx database, GSE16515 cohort and CTPAC database. High expression of HER2 in PDAC was found (Fig. 1A and B), accordant with situations in breast and gastric cancers. Immunostaining was then performed on tissue microarrays from 90 PDAC patients. HER2 was overexpressed (+ +  ~  +  + +) in tumor of 53.3% patients (48/90), higher than that in adjacent normal tissue (Fig. 1C and D). Representative immunostaining of HER2 was shown and the 90 patients were divided into two groups based on the result of IHC analysis: high-HER2-expression group (n = 48) and low-HER2-expression group (n = 42). Accordant with the analysis using TCGA database (Additional file 1: Figure S1A), Kaplan–Meier survival analysis showed that PDAC patients with higher HER2 expression levels did not have worse overall survival (Additional file 1: Figure S1A and B). But patients with higher HER2 expression levels did have worse disease-free survival in TCGA, indicating that HER2 may not act as a main cancer driver-gene in PDAC but still actively participating in the carcinogenesis of PDAC, thus made it an actionable target for clinical treatment. We then performed IHC in 25 PDXs established and found 5 PDXs with high expression of HER2, but none with *HER2* gene amplification tested by FISH analysis, which was reported as a rare issue in PDAC (around 1–7%) [9] HER2 expression in PDXs is shown in Fig. 1E. Taken together, these data suggest that HER2 inhibition by pyrotinib could be explored as an actionable pathway for the treatment of PDAC.

### Pyrotinib monotherapy achieves insufficient anti-tumor effect in PDAC

**Fig. 2:**
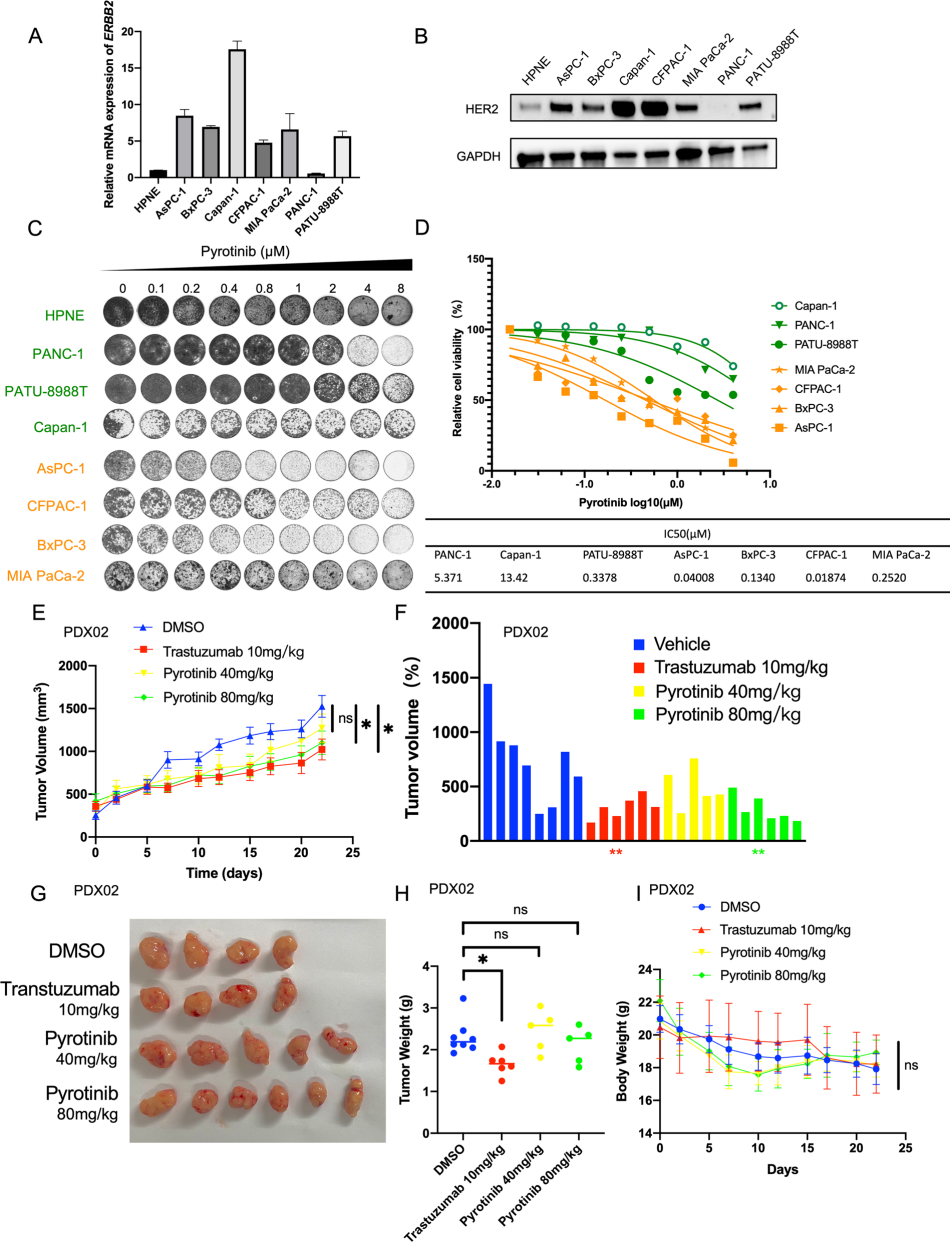
Pyrotinib monotherapy exhibits an insufficient anti-tumor effect in PDAC cell lines in vitro and PDX in vivo. **A**, **B** HER2 expression were exhibited using qPCR and western blot in one immortalized pancreatic ductal cell (HPNE) and seven PDAC cell lines. **C**, **D** PDAC cell lines were treated with increasing concentrations of pyrotinib. Colony formation assay were conducted (**C**) and IC50 were calculated (**D**). **E**–**I** one patient-derived xenograft (PDX02) was planted in BALB/c nude mice and the fifth passage mice were delivered with vehicle (n = 7), trastuzumab (10 mg/kg, intraperitoneal injection; once every two days, n = 5), pyrtonib (40 mg/kg or 80 mg/kg respectively, oral gavage; every day, n = 5 each) for 21 days. Growth curve and endpoint tumor volume were delivered in (**E**) and (**F**–**H**). Body weight curve were delivered in (**I**). Data are represented as mean ± SEM. Statistical significance were assessed using a Student’s t test. *P < 0.05, **P < 0.01, ***P < 0.001, ****P < 0.0001. *ns* not significant

To evaluate the efficacy of pyrotinib monotherapy in PDAC, we firstly examined the mRNA levels of HER2 and detected the protein level of HER2 in 7 PDAC cell lines and 1 immortalized pancreas ductal cell line (HPNE) (Fig. 2A and B). IC50 was calculated in 7 PDAC cell lines, and consistent with expression pattern of HER2, cell lines harboring higher expression of HER2 showed higher sensitivity when treated with pyrotinib except for Capan-1 and PATU-8988 T (Fig. 2D). We then treated the panel of 8 cell lines with increasing concentrations of pyrotinib in long-term colony formation assays and we found that pyrotinib treatment only severely impaired the proliferation of four cell lines including AsPC-1, BxPC-3, CFPAC-1 and MIA PaCa-2 (Fig. 2C). To explore the therapeutic effect of pyrotinib in vivo, mice bearing patient-derived xenografts with high expression of HER2 (PDX02) (Fig. 1E) were delivered with two dosages of pyrotinib (40 mg/kg and 80 mg/kg) and one dosage of trastuzumab (10 mg/kg), the classical HER2 monoclonals as well as vehicle. Compared with trastuzumab, pyrotinib barely showed superiority in suppressing the growth of xenografts (Fig. 2E–I). These data suggest that targeting HER2 by pyrotinib monotherapy is insufficient for PDAC. This urges us to look for potent combination strategies to augment the anti-HER2 efficacy.

### *HDACs/mTOR inhibitor 1 exhibits cytotoxicity combined with pyrotinib *in vitro

**Fig. 3:**
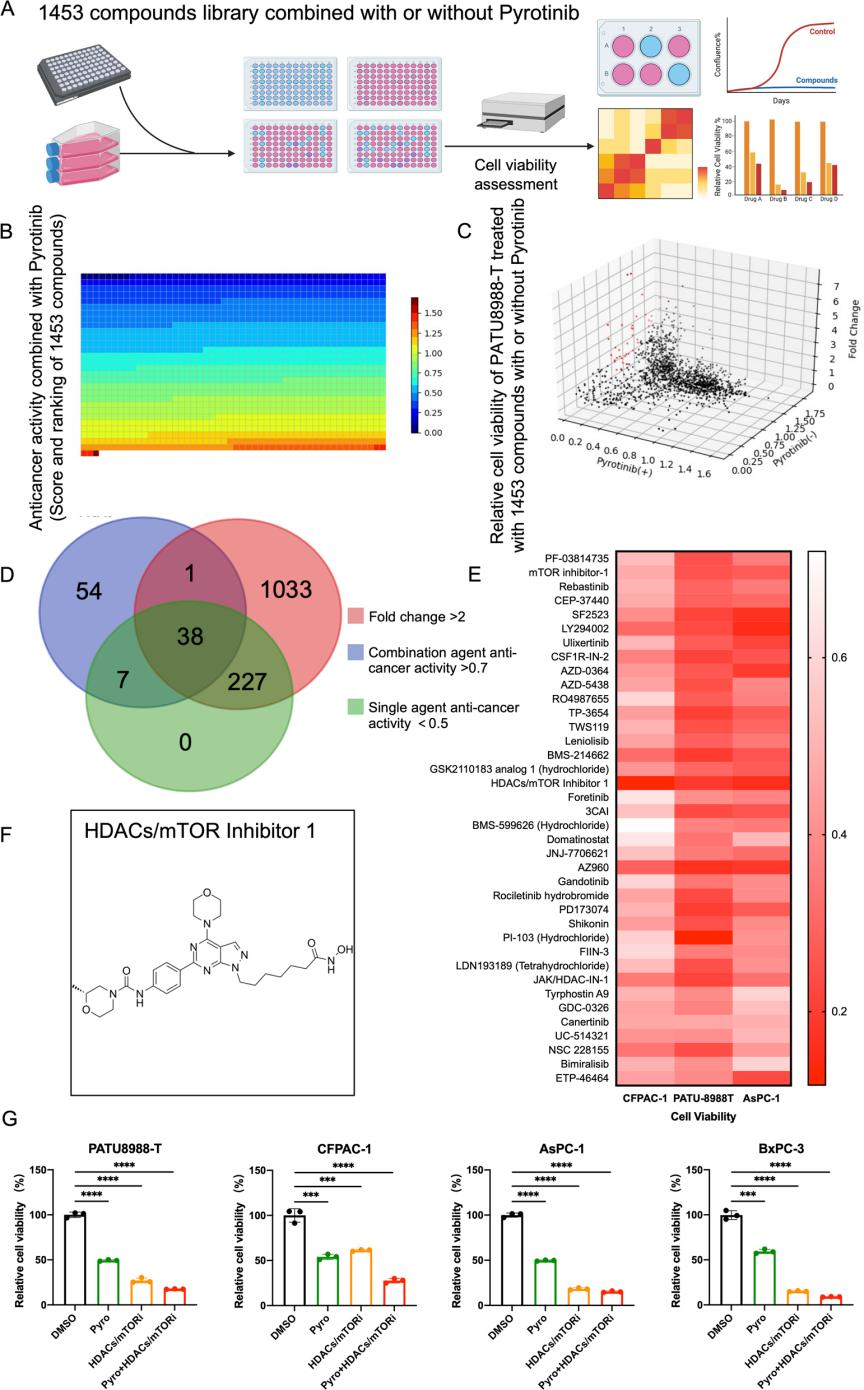
Anticancer activity of HDACs/mTOR inhibitor 1 in vitro. **A** Schematic review of screening of latent compounds for combination in an anti-pancreatic cancer library of 1453 compounds in PATU 8988-T. **B** Cell viability of PATU 8988-T treated with compounds (2 μmol/L) combined with pyrotinib (2 μmol/L) for 72 h. The ranking of the relative anticancer activity is shown by the heatmap; each block represents 1 compound. **C** Single agent anticancer activity (y-axis), combination agent anticancer activity (x-axis) and fold change of x and y (z-axis) were depicted in a 3D coordinate in C and red dots showing compounds content with screening threshold: single agent anticancer activity < 0.5, combination anticancer activity > 0.7 and fold change > 2. **D** Numbers of compounds content with thresholds listed in venn diagram. **E** CFPAC-1 and AsPC-1 as well as PATU 8988-T treated with 38 selected compounds (1 μmol/L) and pyrotinib (1 μmol/L) for 72 h and cell viabilities were calculated. **F** Chemical structure of HDACs/mTOR inhibitor 1. **G** 4 cell liens were treated with HDACs/mTOR inhibitor 1 (0.5 μmol/L), pyrotinib (1 μmol/L) or combination regiments. Data are represented as mean ± SEM. Statistical significance was assessed using a Student t test. *P < 0.05, **P < 0.01, ***P < 0.001. *ns* not significant

To identify latent agents that can synergize with or augment the cytotoxicity of pyrotinib, we screened an anti-pancreatic cancer library of 1453 compounds in the human PDAC cell line PATU8988-T which exhibits high HER2 expression but poor response to pyrotinib in vitro (Fig. 3A). A suitable concentration of 2 μM of pyrotinib was decided at which PATU-8988 T could still survival but was restrained. Aiming to identify the promising agents which may synergize with pyrotinib, we select the agents with the strongest anticancer activity observed when combined with pyrotinib. Fold change were calculated using relative cell viability treated with these compounds between single agent and combination regiments with pyrotinib. To avoid overwhelming single agent efficacy, agents showing anticancer activity > 0.5 were excluded and then 38 compounds were selected which were content with the threshold including fold change > 2 and anticancer activity > 0.7 at the same dosage (Fig. 3B–D). Another two PDAC cell lines CFPAC-1, AsPC-1 were treated with combination of pyrotinib and 38 compounds (Fig. 3E) among which 10 of the most promising agents were testified in a larger scale of dose-combination manners (Additional file 1: Figure S2). Among them HDAC/mTOR Inhibitor1, a novel dual-target histone deacetylase (HDAC) and mammalian target of rapamycin (mTOR) inhibitor (Fig. 3F), shows promising cytotoxicity combined with pyrotinib in 4 PDAC cell lines (Fig. 3G). The promising cytotoxicity exhibited in vitro implicate a latent strategy against PDAC with high expression of HER2 using pyrotinib combined with HDAC/mTOR Inhibitor1.

### HDACs/mTOR inhibitor 1 synergize with pyrotinib and induces apoptosis in PDAC

**Fig. 4:**
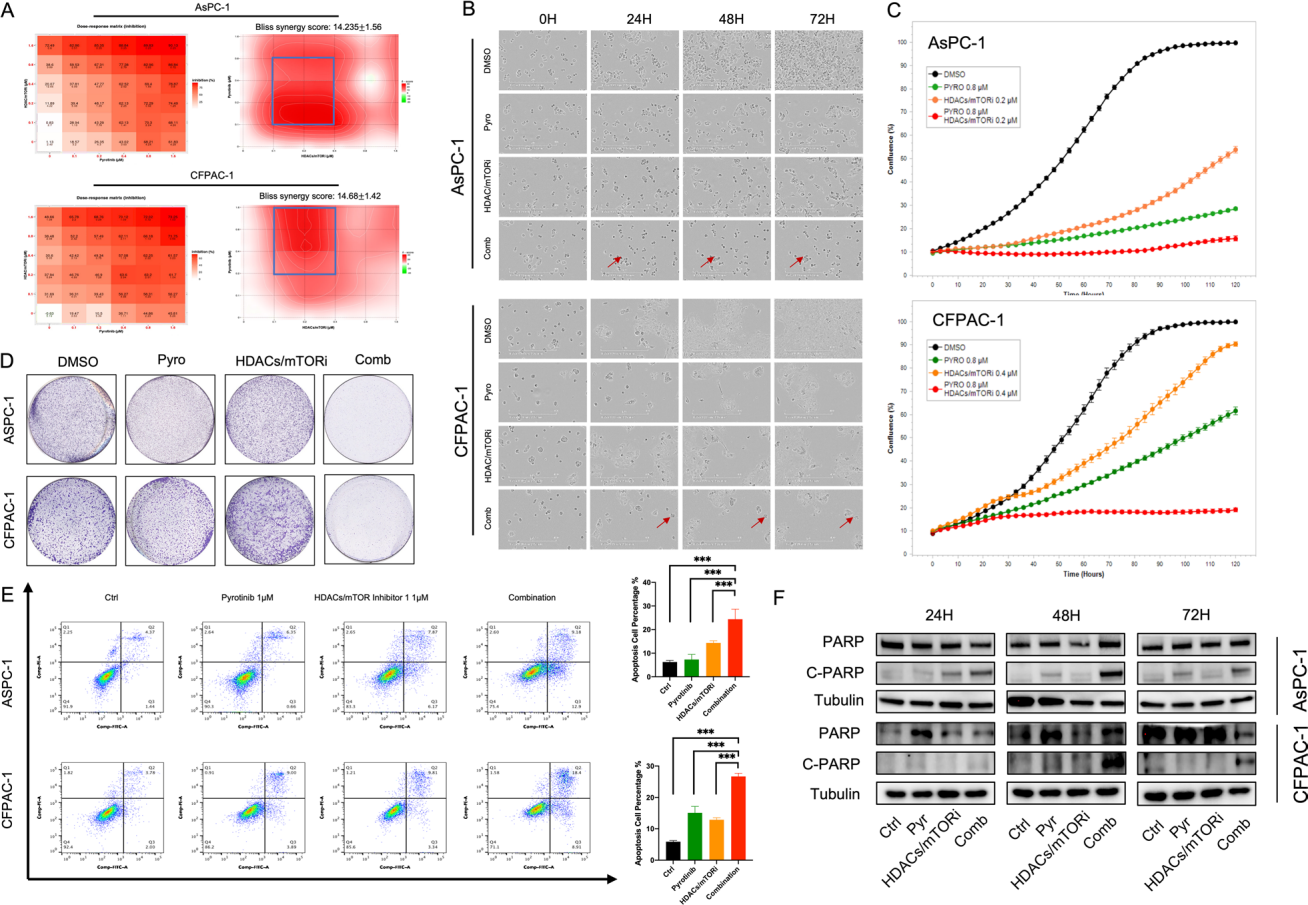
HDACs/mTOR inhibitor 1 synergize with pyrotinib and induces PDAC cells to apoptosis in vitro. **A** Dose–response matrix of inhibition rates of AsPC-1 and CFPAC-1 treated with dosages of pyrotinib (0, 0.1, 0.2, 0.4, 0.8, 1.6 μM) or HDACs/mTOR inhibitor 1 (0, 0.1, 0.2, 0.4, 0.8, 1.6 μM); most of the region in the right block showing Bliss synergy score surpass 10, meaning synergistic effect. **B** Representative pictures of cells underwent apoptosis or not during 72 h; AsPC-1 and CFPAC-1 were treated with DMSO, 0.8 μM of pyrotinib, 0.2 μM(AsPC-1) or 0.4 μM(CFPAC-1) of HDACs/mTOR inhibitor 1 or the combination, arrows showing morphology of apoptosis initiated within 24 h. **C** Proliferation curves of AsPC-1 and CFPAC-1 treated with monotherapy and combination for 120 h. Cell numbers were indirectly indicated using confluence. **D** Colony formation assay of AsPC-1 and CFPAC-1 treated with monotherapy and combination for 7 days. **E** Apoptosis analysis in AsPC-1 and CFPAC-1; cells treated with pyrotinib, HDACs/mTOR inhibitor 1 or combinations were stained with Annexin V/PI. **F** Western blot of cells treated with pyrotinib, HDACs/mTOR inhibitor 1 or combinations with PARP and cleavage PARP. Data are represented as mean ± SD. Statistical significance was assessed using a Student t test. *P < 0.05, **P < 0.01, ***P < 0.001. *ns* not significant

To elucidate the anti-cancer activity in PDAC of HDACs/mTOR inhibitor 1 and pyrotinib, we firstly assessed whether it had synergy effect with pyrotinib. AsPC-1 and CFPAC-1 cells were treated with HDACs/mTOR inhibitor 1 and pyrotinib at a series of dosages and cell viability was decided using CCK-8. With dose–response matrix depicted using Synergyfinder, a stand-alone application for interactive analysis of drug combination screening data [22], median combination indexes were calculated using Bliss synergy score, which was defined as synergy (14.235 and 14.68 in AsPC-1 and CFPAC-1) when > 10 (Fig. 4A). In the right block, regions with most profound synergy effect lies in AsPC-1 with dose of pyrotinib 0.2 to 0.8 μM and HDACs/mTOR inhibitor 1 0.1 to 0.4 μM while in CFPAC-1 with dose of pyrotinib 0.2 to 1.6 μM and HDACs/mTOR inhibitor 1 0.1 to 0.4 μM. To evaluate the synergistic toxicity in PDAC of the combination therapy apart from cell viability assay, we treated AsPC-1 and CFPAC-1 with pyrotinib at a dose of 1 μM, or HDACs/mTOR inhibitor 1 at a dose of 0.5 μM or combination, compared with DMSO. The combination treatment led to a dramatic suppression of proliferation and induced obvious cell apoptosis compared with monotherapy or control which initiates in the first 24 h (Fig. 4B–D). AsPC-1 and CFPAC-1 cells received combination treatment underwent a larger-scale of apoptosis compared with single agent using flow cytometry analysis (Fig. 4E) and verified by western blotting with a sharp increase of cleavage PARP (Fig. 4F). Collectively, these data demonstrate the synergistic toxicity of HDACs/mTOR inhibitor 1 combined with pyrotinib in PDAC and induce apoptosis effect in vitro.

### HDACs/mTOR inhibitor 1 combined with pyrotinib suppresses the growth of CDX and PDX of PDAC

**Fig. 5:**
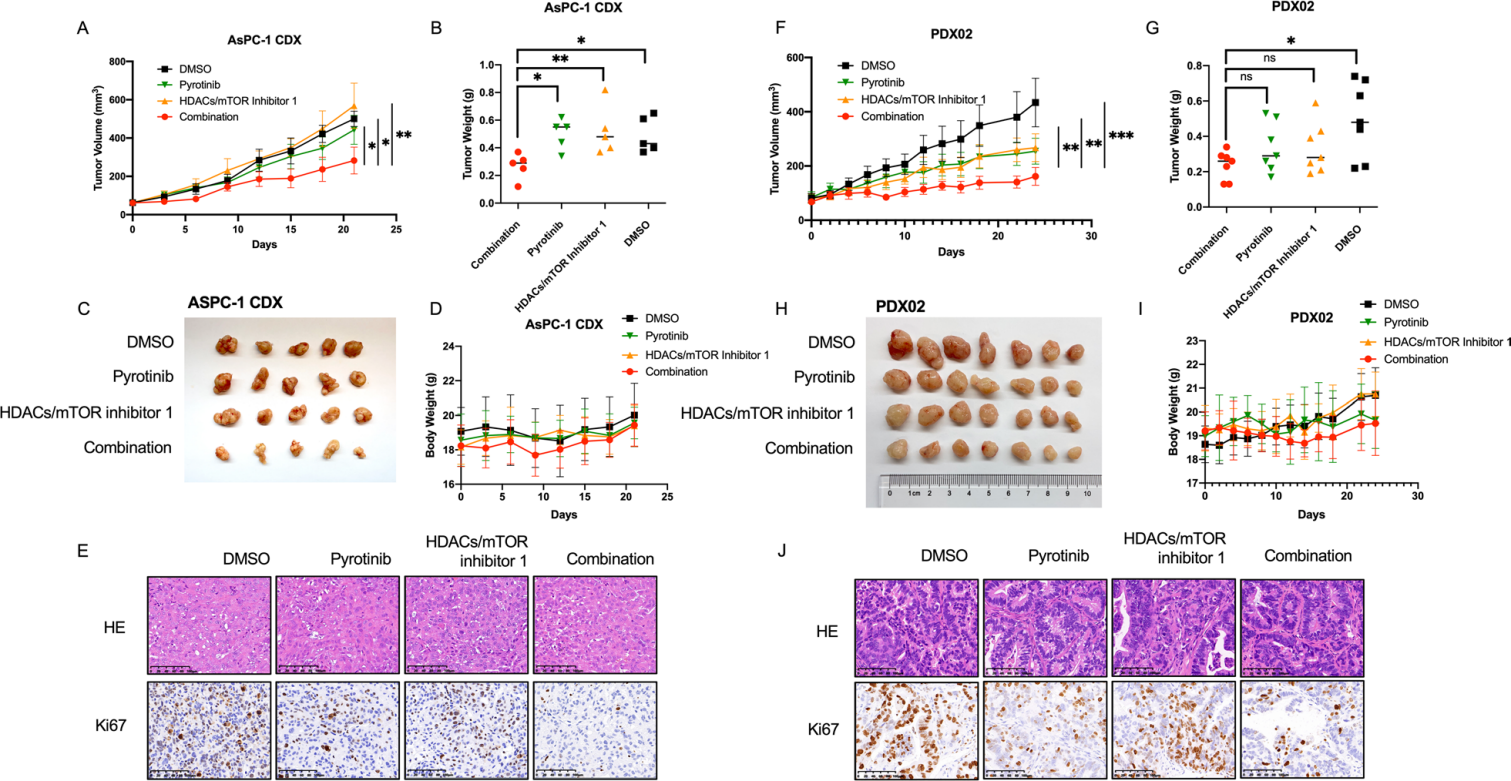
HDACs/mTOR inhibitor 1 combined with pyrotinib suppresses the growth of CDX and PDX of PDAC. (A-D, F-I) Cell-deived xenografts of AsPC-1 and patient-derived xenografts from PDAC tumors were treated with vehicle, pyrotinib 40 mg/kg, HDACs/mTOR inhibitor 1 10 mg/kg or combined once the tumors reached 50-100mm^3^ and randomized for 21 days. The tumor volume and body weight were monitored every 2–3 day. Curves of tumor volume were shown in (**A**) and (**F**), weight of tumors was shown in (**B**) and (**G**), pictures of tumors were shown in (**C**) and (**H**), curves of body weight were shown in (**D**) and (**I**). **E**, **J** Identical pictures of H&E and IHC staining of Ki67 were shown in (**E**) and (**J)**. Scale bars, 100 μM. Data are represented as mean ± SD. Statistical significance was assessed using a Student t test. *P < 0.05, **P < 0.01, ***P < 0.001. *ns* not significant

To assess whether in vitro findings can be recapitulated in vivo, we established subcutaneous tumors in nude mice using AsPC-1 cells and PDX02. When tumor grew to an average size around 50-100mm^3^, mice were randomized into four groups treated with pyrotinib 40 mg/kg, HDACs/mTOR inhibitor 1 10 mg/kg or combined with same dosages of vehicle. Compared with groups received pyrotinib or HDACs/mTOR inhibitor 1 monotherapy or vehicle, combination group had obviously stronger antitumor effect in CDXs and PDXs with significant reduction of tumor volume and weight (Fig. 5A–D, F–I). Compared with vehicle and monotherapy treated tumors, tumor tissues isolated from mice treated with combination therapy had reduced proliferation, as indicated by immunostaining of Ki67 in both CDXs and PDXs (Fig. 5E, J). These data shows in vivo anti-PDAC efficacy of the combination therapy in vivo.

### HDAC inhibition mediates the degradation of mutant P53 to promote apoptosis together with HER2 and mTOR inhibition in PDAC

**Fig. 6:**
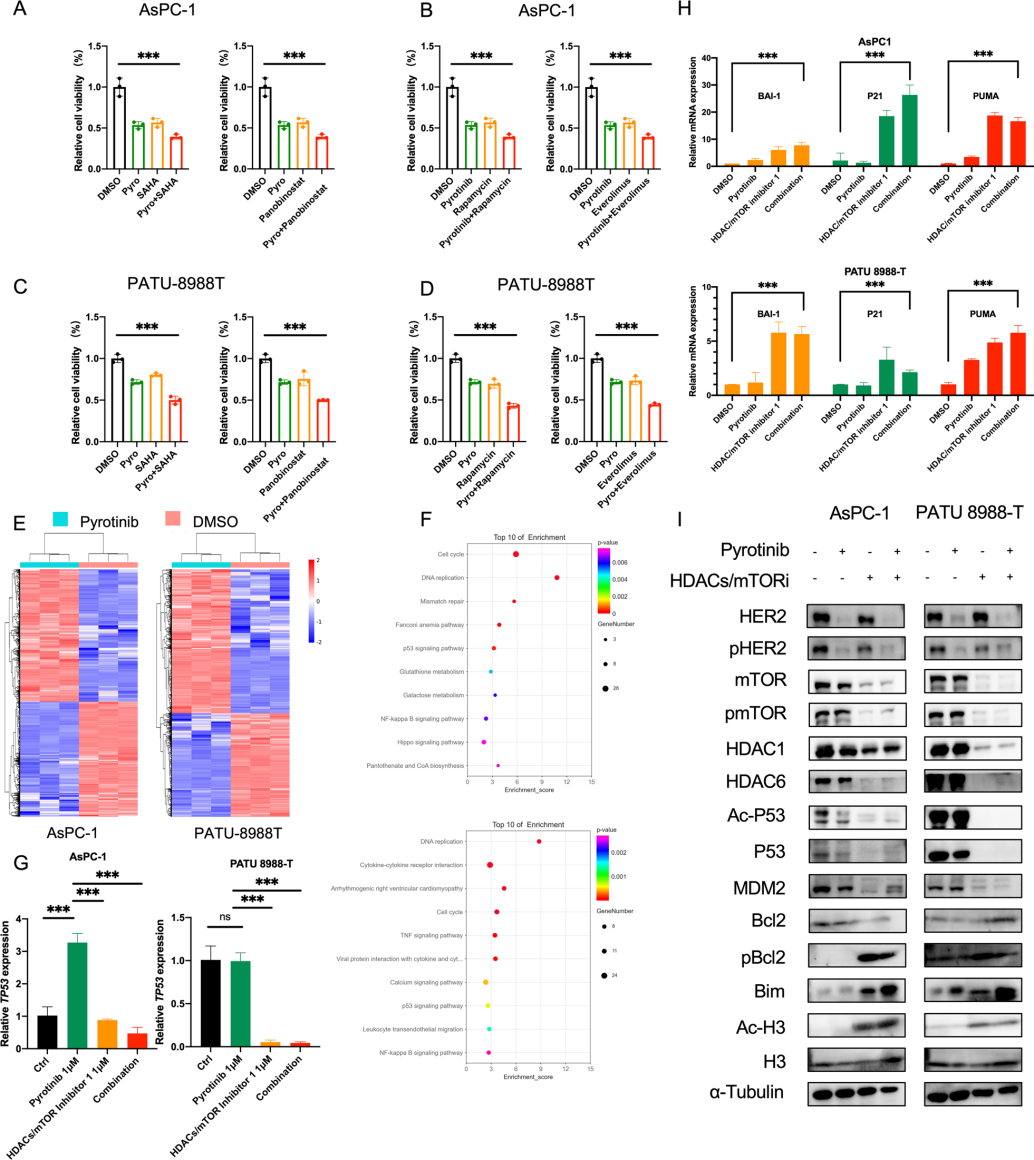
HDAC inhibition mediates the degradation of mutant P53 to promote apoptosis together with HER2 and mTOR inhibition in PDAC. **A**–**D** Cells were treated with DMSO or pyrotinib of 1 μM or HDAC inhibitor (SAHA of 2 μM, Panobinastat of 5 nM) or mTOR inhibitors (Rapamycin of 15 μM, Everolimus of 20 μM) or combined. Cell viability was assessed by CCK8. **E** Heatmap of gene expression values in AsPC-1 and PATU 8988-T treated with pyrotinib versus DMSO for 24 h. **F** Top 10 of enrichment score for selected GO functional catagories of top 10% differential expressed genes in AsPC-1 and PATU 8988-T. **G** Validation of P53 by quantitative PCR analysis in AsPC-1 and PATU 8988-T. **H** Downstream BAI1, P21 and PUMA analysed by quantitative PCR analysis in AsPC-1 and PATU 8988-T. **I** Immunoblo analysis of the HER2, pHER2, mTOR, pmTOR, HDAC1, HDAC6 as direct targets of combination therapy and acetylation of P53 and H3 as well as P53 and its downstream MDM2 Bcl2 and pBcl2 and Bim in AsPC-1 and PATU 8988-T cells treated with DMSO, monotherapy or combination therapy. Data are represented as mean ± SD. Statistical significance was assessed using a Student t test. *P < 0.05, **P < 0.01, ***P < 0.001. ns, not significant

Next, we south to clarify whether HDAC inhibition and/or mTOR inhibition is synergistic with HER2 inhibition. AsPC-1 and PATU-8988 T were treated with two well recognized pan-HDAC inhibitors (SAHA and panobinostat) and mTOR inhibitors (rapamycin and everolimus) combined with or without pyrotinib. Cell viability of combination group was significantly hampered both in HDACi-treated group and mTORi-treated group (Fig. 6A–D), indicating both anti-HDACs and anti-mTOR synergized with pyrotinib in PDAC. The synergistic effect of the combination of anti-HER2 and its downstream mTOR signaling pathway had been explained by the upregulation of HER2 signaling in response to mTOR inhibition [23]. Then we focused on the exploration of latent molecular mechanisms of how HDAC inhibition synergizes with pyrotinib. AsPC-1 and PATU-8988 T cells treated with pyrotinib or DMSO were assessed at transcriptome levels using RNA sequencing experiments (Fig. 6E). Top 10% differential expressed genes were subjected to GO enrichment analysis and we found genes involved in DNA replication and p53 signaling pathway were preferentially up- or down-regulated both in AsPC-1 and PATU-8988 T (Fig. 6F), indicating that pyrotinib treatment rendered distinct DNA replication stress and corresponding P53 signaling alternation. As reported, apart from histone targets, histone deacetylase involves in regulation of a growing number of non-histone targets such as P53, E2F1, STAT1 and NF- kB [24]. HDAC inhibition can induce wild-type P53 hyperacetylation that stabilizes and transcriptionally activates P53 for pro-apoptotic targets. Meanwhile, as in a double-blade sword manner, mutant P53 was reported to be destabilized treated with HDAC inhibition through inhibition of the HDAC6-Hsp90 chaperone axis. mRNA level of P53 and its downstream genes including BAI1, PUMA, P21 that involving in pro-apoptosis were significantly downregulated or upregulated in combination treatment compared with monotherapy or DMSO treatment (Fig. 6G and H). Further validation in protein level was conducted as shown in Fig. 6I. Both acetylated P53 and P53 were significantly degraded with its downstream protein MDM2 and Bcl2 down-regulated and Bim pathway were activated both in AsPC-1 and PATU 8988-T, indicating an active involvement of mutant P53 in the combination therapy. Taken together, these results indicate the mutant P53 were degraded with HDAC inhibition and thus, together with HER2 and mTOR inhibition, inducing apoptosis in PDAC.

## Discussion

The role of targeting HER2 in PDAC treatment is yet to be established despite its success in treatment of a variety of different cancers such as breast cancer, gastric cancer and lung cancer [25]. Two clinical trials have assessed targeted HER2 therapy in PDAC using trastuzumab, combined with gemcitabine or capecitabine [26, 27]. The addition of trastuzumab to chemotherapy did not improve OS nor PFS in metastatic PDAC. In this study we evaluated the efficacy of pyrotinib, a pan-HER tyrosine kinase inhibitor that irreversibly blocks EGFR, HER2 and HER4, in HER2-positive PDAC. We found 53.3% patients in our cohort have high expression of HER2 and that pyrotinib inhibited cell proliferation in HER2-positive PDAC cell lines and PDX but struggled in inducing cell death. Combined drug treatments aiming to augment the toxicity and hamper the drug resistance is thus to be explored.

Here we reported HDACs/mTOR inhibitor 1, a dual target inhibitor which showed potent synergistic effect with pyrotinib against PDAC both in vitro and vivo using large-scale compound library screening. HER2 signaling is primarily mediated through downstream PI3K/Akt and MAPK axis which govern cell proliferation and apoptosis. The importance of the PI3K/Akt and MAPK pathways in oncogenic signaling is becoming increasingly apparent. PI3K/Akt/mTOR signaling pathway is potential targets for HER2 + cancer with drug resistance considering the anticipated synergy effect. Clinical trials have suggested HER2-directed therapies in combination with agents targeting PI3K signaling. For example, combinations of trastuzumab or lapatinib with everolimus, a mTOR inhibitor, have demonstrated encouraging antitumor activity in HER2-overexpressing tumors [28, 29]. We detected the anticipated synergistic effect of HER2 and mTOR inhibition and the effect of HER2 and HDACs (specifically HDAC1 and HDAC6) inhibition. As a promising class of anti-cancer targets, HDAC involves in regulating expression of a ground amount of genes including pro-apoptotic genes, the epithelial marker E-cadherin, genes promoting neoplastic cell or cell differentiation and those involved in DNA damage responses, thus showing synergistic cytotoxicity with several targeted therapies including EGFR [30].

In this study, we detected strong synergistic effect of pyrotinib and HDACs/mTOR inhibitor 1 using Synergyfinder, and we found both mTOR and HDAC inhibition augment cytotoxic of pyrotinib in PDAC cells. Mechanically, we found distinct change of P53 pathway related genes in cells treated with pyrotinib. Expression of P53 might relate with the limited anti-proliferation effect of pyrotinib in PDAC. All pancreatic cancer cell lines used in this study harbor P53 mutation as shown in Additional file 1: Table S4. Previous studies indicated that gain-of-function of mutant P53 in pancreatic cancer involved in the malignant progression and drug resistance, including activating oncogenic RAS signaling [19]. Mutant P53 was reported to enhance the transcription level of HER2 and further stabilize it through HSP90 [31]. SAHA, the classical HDAC inhibitor approved by FDA exhibits preferential cytotoxicity for mutant rather than wild-type P53 human cancer cells. Furthermore, SAHA’s inhibition of HDAC6 which was an essential positive regulator of HSP90, was validated as the mediator of mutant P53 degradation [21]. Other studies focusing on HDAC1 reported that inhibition of HDAC1 mediated acetylation of mutant P53 in pancreatitis, and thus to restore the pro-apoptosis function of P53 [32]. In our study, we verified the specifically inhibition of HDAC1 and HDAC6 in cells treated with pyrotinib and HDACs/mTOR inhibitor 1 and the distinct degradation of both P53 and its acetylated homolog. These results indicated that HDACs/mTOR inhibitor 1 may synergized with pyrotinib in PDAC through mediating the degradation of mutant P53 by inhibition of HDAC6 as well as mTOR. However, gain- or loss-of-function of P53 had not be validated in our study in the condition of silence of HDAC6 which needed further exploration.

Over all our data identified a novel mutant P53 directed synergy effect of pyrotinib and HDACs/mTOR inhibitor 1 and proposed the rationale and necessity to next test of HER2 and HDACs inhibition in more preclinical experiment and ambitiously in future clinical studies.

## Supplementary Information

**Additional file 1:** Extanded materials and methods.** Figure S1**.Clinical significance of HER2 in TCGA and Renji cohorts.  **Figure S2.** Different dose-combination of pyrotinib and top 10 compounds showing the augment effect of HDACs/mTOR inhibitor 1(left panel top) combined with pyrotinib in 4 cell lines. **Table S1.** Summary of HER2 targeted drugs. **Table S2**. Targets and related pathway of Top 38 drug screened showing tocixity combined with Pyrotinib. **Table S3. **Q-PCR primers used in this study. **Table S4. **Mutant status of *P53* in PDAC cell lines in this study.

## Data Availability

All data needed to evaluate the conclusions in the paper are present in the paper and/or the Additional file Materials. The accession number for the publicly available microarray dataset analyzed in this study are accessible through the GEO series accession numbers GSE16515. Other data that support the findings of this study are available from the corresponding author on reasonable request.
