# Supplementary material for: HDACs/mTOR inhibitor synergizes with pyrotinib in HER2-positive pancreatic cancer through degradation of mutant P53
Source: Cancer Cell Int. 2022 Dec 1;22:380. doi: 10.1186/s12935-022-02807-4 (PMC9714091; doi:10.1186/s12935-022-02807-4)
Supplement: Supplementary file 1 — Additional file 1: Extanded materials and methods. Figure S1.Clinical significance of HER2 in TCGA and Renji cohorts. Figure S2. Different dose-combination of pyrotinib and top 10 compounds showing the augment effect of HDACs/mTOR inhibitor 1(left panel top) combined with pyrotinib in 4 cell lines. Table S1. Summary of HER2 targeted drugs. Table S2. Targets and related pathway of Top 38 drug screened showing tocixity combined with Pyrotinib. Table S3. Q-PCR primers used in this study. Table S4. Mutant status of P53 in PDAC cell lines in this study. [file 12935_2022_2807_MOESM1_ESM.docx]

Additional file materials

1. Extended Materials and methods

1.1. Cell culture and reagents

Human PDAC cell lines AsPC-1, BxPC-3, Capan-1, CFPAC-1, Mia PaCa-2, PANC-1, PATU-8988T and human normal pancreatic duct cell (HPNE) all preserved in Shanghai Cancer Institute, Renji Hospital, School of Medicine, Shanghai Jiao Tong University. Cells were grown in suggested culture medium, supplemented with 10% (or 20% with Capan-1) fetal bovine serum (FBS, Gibco), 100U/mL penicillin and 100 μg/mL streptomycin at 37℃ with 5% CO2 condition. The base medium for AsPC-1, BxPC-3, PATU-8988T, MIA PaCa-2 is RPMI-1640 Medium, for Capan1 and CFPAC-1 is Iscove's Modified Dulbecco's Medium (IMDM), for PANC-1 is Dulbecco's Modified Eagle's Medium (DMEM), for MIA PaCa-2 is DF12 Medium. All cell lines underwent verification by Shanghai Cancer Institute and regular testing (every 4 months) to ensure lack of contamination with the Mycoplasma. The number of passages between thawing cell lines and their use in the described experiments was 2-30.

Pyrotinib and Trastuzumab were obtained from Selleck (Texas, USA). HDACs/mTOR inhibitor 1 and Compound libraries were purchased from MCE (Shanghai, China). HDAC inhibitors (SAHA and panobinostat) and mTOR inhibitors (rapamycin and everolimus) was purchased from MCE (Shanghai, China). Cell Signaling Technology (Massachusetts, USA).

1.2. Cell viablitiy assay

Cells with indicated treatment were grown in 96-well plate at 3,000 cells /well, which were performed in triplicate. At the indicated time point, CCK-8 was added into the well and incubated 1 hour at 37℃. Absorbance at 450nm was measured by microplate reader (M1000 PRO, TECAN). The experiments were performed in triple manner and repeated twice.

1.3. Colony formation assays

In brief, 3,000 PDAC cells suspended in 2 mL of complete medium were seeded into 6-well plates after treatment with indicated conditions. Culture medium was replaced every week. The cells were allowed to grow for the next 2 weeks to allow colony formation and the colonies were visualized with crystal violet staining. This experiment was repeated twice.

1.4. RNA isolation and gene expression study

Total RNA extraction and RNA reversely transcription are using Trizol reagent (Takara, 9109) and PrimeScript RT-PCR kit (Takara, RR037A) according to the common protocols. Real-time PCR analyses were applied for gene expression study and performed with SYBR Premix Ex Taq (Roche, 04913914001) was used to run PCR on a 7500 Real-time PCR system (Applied Biosystems) at the recommended thermal settings. Relative mRNA expression was calculated using the 2(-ΔΔCt) method and normalized to18s mRNA levels. Primer sequences are listed in Additional file Table 2.

1.5. RNA sequencing

Total RNAs of cell samples were isolated using the TRIzol reagent for RNA sequencing following the manufacturer’s instructions. Their qualities were checked using an Agilent 2100 Bioanalyzer (Agilent). The library fragments were purified with AMPure XP system (Beckman Coulter, Beverly, USA). The clustering of the index-coded samples was performed on a cBot Cluster Generation System using TruSeq PE Cluster Kit v3-cBot-HS (Illumia) according to the manufacturer’s instructions. After cluster generation, the library preparations were sequenced on an Illumina Hiseq X Ten and 150 bp paired-end reads were generated. HTSeq v0.6.0 was used to count the reads numbers mapped to each gene. And then FPKM of each gene was calculated based on the length of the gene and reads count mapped to this gene.

1.6. Immunoblotting

Cell lysates were prepared by using protein extraction buffer (Sangon, Shanghai, China), followed centrifugation in 4 ℃ for 10 min and collected supernatant. BCA Protein Assay kit (Pierce Biotechnology) was utilized to measured protein concentration. After total protein normalized, protein samples were separated by 8-10% SDS-PAGE gel electrophoresis and transferred onto 0.45 μm NC membranes (Whatman, GE). After blocking with 5% skimmed milk or 3 % BSA diluted in Tris buffer saline plus 0.1% Tween 20 (TBST) for 1hr at room temperature, membranes were incubated overnight at 4℃ with the following primary antibodies: HER2 (1:1000,CST, 2165), pHER2 (1:1000,CST, 6924), PARP (1:1000,CST, 3542), c-PARP (1:1000,CST, 5625), alpha-tubulin (1:1000,CST, 2125), mTOR (1:1,000, CST, 2983), p-mTOR (1:1,000, CST, 2971), HDAC1 (1:1,000, CST, 34589), HDAC6 (1:1,000, CST, 7558), P53 (1:1,000, CST, 2527), Ac-P53 (1:1,000, CST, 2525), MDM2 (1:1,000, CST, 86934), Bcl2 (1:1,000, CST, 4223), pBcl2 (1:1,000, CST, 2827), Bim (1:1,000, CST, 2971), H3 (1:1,000, CST, 4499), Ac-H3 (1:1,000, CST, 4243),Then membranes were washed three times of 10 min each time in TBST at room temperature, and incubated with goat anti-mouse(1:10,000, Jackson ImmunoResearch, 115-035-003) or rabbit secondary antibodies (1:10,000) Jackson ImmunoResearch, 111-035-003) for 1 hr at room temperature. Enhanced Chemiluminiscence (ECL) was performed using ECL kit (WB012, share-bio, China), visualized by the Bio-Rad system.

1.7. Histology and Immunohistochemistry

Hematoxylin and eosin (H&E) staining was performed according to standard methods. For immunohistochemical staining, firstly slides were deparaffinized in xylene. Next, slides were incubated with 0.3% hydrogen peroxide in methanol to block endogenous peroxidase. Antigen retrieval was performed by boiling the slides in pH 6.0 citrate buffer for 10 min. After blocked for 1 hr at room temperature with 10% BSA ,slides were immunostained using antibodies against Ki67 (1:800, CST, 9449). Following 1hr incubation with HPR-conjugated secondary antibody (anti-mouse, 1:500, Jackson ImmunoResearch, 115-035-003 or rabbit secondary antibodies 1:500 Jackson ImmunoResearch, 111-035-003), slides were developed in DAB (CST, 8059) and counterstained with hematoxylin.

1.8. Fluorescence insitu hybridization

HER2 testing was performed in the clinical laboratory improvement amendments laboratories at MSK and UCCC. HER2 amplification was assessed by fluorescence in situ hybridization (FISH) using U.S. Food and Drug Administration–approved probe sets (PathVysion [Abbott] and HER2 IQFISH pharmDx [Dako]) and defined as an HER2-to-chromosome enumeration probe 17 (CEP17) ratio of at least 2.0, as used in clinical trials8, 9 (Fig. 1). HER2 mutation was assessed by fragment analysis, mass spectrometry genotyping, and Sanger sequencing for indels in exon 20. In the cases in which tissue was available, HER2 protein overexpression was assessed by IHC staining using the 4B5 Ventana antibody and defined as 2+ or 3+ on the basis of published methods identical to those used for breast cancers.4, 9 In both laboratories, each test was reviewed independently by two experts.

2. Additional file Figures:

**Additional file figure 1.** Clinical significance of HER2 in TCGA and Renji cohorts. **(A)** Kaplan-Meier analysis for OS (left) and DFS (right) in TCGA according to HER2 level. **(B)** Kaplan-Meier analysis for OS in Renji cohort according to HER2 level. **(C)** Multivariate Cox regression analysis of clinicopathologic factor for OS applied in Renji cohort. **(D)** Representative images of HER2 non-amplification in PDXs, fluorescence in situ hybridization using a HER2 IQFISH pharmDxTM probe, Dako (HER2 = red signal, CEP17 = green signal). OS, overall survival; DFS, disease-free survival; CI, confidence interval.

**Additional file figure 2.** Different dose-combination of pyrotinib and top 10 compounds showing the augment effect of HDACs/mTOR inhibitor 1(left panel top) combined with pyrotinib in 4 cell lines.

**Additional file table 1.** Summary of HER2 targeted drugs

| **Drug** | **Brand Name** | **Monoclonal Antibodies** | **Route of Administration** | **TKIs** | **Antibody-drug Conjugate** | **Target** | | | |
| --- | --- | --- | --- | --- | --- | --- | --- | --- | --- |
|  |  |  |  |  |  | **HER1** | **HER2** | **HER3** | **HER4** |
| Trastuzumab | Herceptin | √ | Injection |  |  |  | + |  |  |
| Pertuzumab | Perjeta | √ | Injection |  |  |  | + |  |  |
| Trastuzumab emtansine | Kadcyla |  | Injection |  | √ |  | + |  |  |
| Lapatinib | Tykerb |  | Oral | √ |  | + | + |  |  |
| Neratinib | Nerlynx |  | Oral | √ |  | + | + |  | + |
| Pyrotinib | Irene |  | Oral | √ |  | + | + |  | + |

**Additional file table 2**. Targets and related pathway of Top 38 drug screened showing tocixity combined with Pyroti

| **Product Name** | **Target** | **Pathway** |
| --- | --- | --- |
| BMS-214662 | Farnesyl Transferase | Metabolic Enzyme/Protease |
| JAK/HDAC-IN-1 | HDAC; JAK | Cell Cycle/DNA Damage; Epigenetics; JAK/STAT Signaling; Stem Cell/Wnt |
| Foretinib | c-Met/HGFR; VEGFR | Protein Tyrosine Kinase/RTK |
| FIIN-3 | EGFR; FGFR | JAK/STAT Signaling; Protein Tyrosine Kinase/RTK |
| Shikonin | Chloride Channel; HIV; NF-κB; Pyruvate Kinase; TNF Receptor | Anti-infection; Apoptosis; Membrane Transporter/Ion Channel; Metabolic Enzyme/Protease; NF-κB |
| PF-03814735 | Aurora Kinase; VEGFR | Cell Cycle/DNA Damage; Epigenetics; Protein Tyrosine Kinase/RTK |
| mTOR inhibitor-1 | Autophagy; mTOR | Autophagy; PI3K/Akt/mTOR |
| Tyrphostin A9 | Influenza Virus; VEGFR | Anti-infection; Protein Tyrosine Kinase/RTK |
| Rebastinib | Apoptosis; Bcr-Abl; FLT3; Src | Apoptosis; Protein Tyrosine Kinase/RTK |
| 3CAI | Akt | PI3K/Akt/mTOR |
| BMS-599626 (Hydrochloride) | EGFR | JAK/STAT Signaling; Protein Tyrosine Kinase/RTK |
| LDN193189 (Tetrahydrochloride) | TGF-β Receptor | TGF-beta/Smad |
| Domatinostat | Apoptosis; HDAC | Apoptosis; Cell Cycle/DNA Damage; Epigenetics |
| JNJ-7706621 | Apoptosis; Aurora Kinase; CDK | Apoptosis; Cell Cycle/DNA Damage; Epigenetics |
| GDC-0326 | PI3K | PI3K/Akt/mTOR |
| AZ960 | Apoptosis; JAK; Parasite; Virus Protease | Anti-infection; Apoptosis; Epigenetics; JAK/STAT Signaling; Stem Cell/Wnt |
| Gandotinib | FGFR; FLT3; JAK; VEGFR | Epigenetics; JAK/STAT Signaling; Protein Tyrosine Kinase/RTK; Stem Cell/Wnt |
| CEP-37440 | ALK; FAK | Protein Tyrosine Kinase/RTK |
| SF2523 | DNA-PK; Epigenetic Reader Domain; PI3K | Cell Cycle/DNA Damage; Epigenetics; PI3K/Akt/mTOR |
| NSC 228155 | EGFR; Epigenetic Reader Domain; Histone Acetyltransferase | Epigenetics; JAK/STAT Signaling; Protein Tyrosine Kinase/RTK |
| LY294002 | Apoptosis; Autophagy; Casein Kinase; DNA-PK; PI3K | Apoptosis; Autophagy; Cell Cycle/DNA Damage; PI3K/Akt/mTOR; Stem Cell/Wnt |
| HDACs/mTOR Inhibitor 1 | Apoptosis; HDAC; mTOR | Apoptosis; Cell Cycle/DNA Damage; Epigenetics; PI3K/Akt/mTOR |
| PI-103 (Hydrochloride) | Apoptosis; Autophagy; DNA-PK; mTOR; PI3K | Apoptosis; Autophagy; Cell Cycle/DNA Damage; PI3K/Akt/mTOR |
| Rociletinib hydrobromide | EGFR | JAK/STAT Signaling; Protein Tyrosine Kinase/RTK |
| Bimiralisib | mTOR; PI3K | PI3K/Akt/mTOR |
| Ulixertinib | ERK | MAPK/ERK Pathway; Stem Cell/Wnt |
| CSF1R-IN-2 | c-Fms; c-Met/HGFR; Src | Protein Tyrosine Kinase/RTK |
| AZD-0364 | ERK | MAPK/ERK Pathway; Stem Cell/Wnt |
| AZD-5438 | CDK | Cell Cycle/DNA Damage |
| PD173074 | Apoptosis; FGFR; VEGFR | Apoptosis; Protein Tyrosine Kinase/RTK |
| RO4987655 | MEK | MAPK/ERK Pathway |
| Canertinib | EGFR | JAK/STAT Signaling; Protein Tyrosine Kinase/RTK |
| TP-3654 | Pim | JAK/STAT Signaling |
| UC-514321 | Apoptosis; STAT | Apoptosis; JAK/STAT Signaling; Stem Cell/Wnt |
| ETP-46464 | ATM/ATR; mTOR | Cell Cycle/DNA Damage; PI3K/Akt/mTOR |
| TWS119 | Autophagy; GSK-3 | Autophagy; PI3K/Akt/mTOR; Stem Cell/Wnt |
| Leniolisib | PI3K | PI3K/Akt/mTOR |
| GSK2110183 analog 1 (hydrochloride) | Akt | PI3K/Akt/mTOR |

**Additional file table 3.** Q-PCR primers used in this study

| **Gene symbol** | **Forward sequence (5’-3’)** | **Reverse sequence (5’-3’)** |
| --- | --- | --- |
| *18S* | TGCGAGTACTCAACACCAACA | GCATATCTTCGGCCCACA |
| *P53* | CCTCAGCATCTTATCCGAGTGG | TGGATGGTGGTACAGTCAGAGC |
| *BAI1* | ACAACCTGGTTCTCAGCATCC | GGACGGTCGTGTTCCTCTG |
| *P21* | CGATGGAACTTCGACTTTGTCA | GCACAAGGGTACAAGACAGTG |
| *PUMA* | ACGACCTCAACGCACAGTACGA | CCTAATTGGGCTCCATCTCGGG |

**Additional file table 4.** Mutant status of *P53* in PDAC cell lines in this study

| **Cell line** | **TP53 variant type** | **Protein Change** |
| --- | --- | --- |
| *AsPC-1* | DEL | C135fs |
| *BxPC-3* | SNP | Y220C |
| *CFPAC-1* | SNP | C242R |
| *CaPan 1* | SNP | A159V |
| *PANC1* | SNP | R273H |
| *Mia PaCa-2* | SNP | R248W |
| *PATU 8988-T* | SNP | NA |
